# Hepatitis B virus core antigen mutations predict post-operative prognosis of patients with primary hepatocellular carcinoma

**DOI:** 10.1099/jgv.0.000790

**Published:** 2017-06-22

**Authors:** Jian’an Jia, Huiming Li, Hui Wang, Shipeng Chen, Mengmeng Wang, Huijuan Feng, Yuzhen Gao, Yunjiu Wang, Meng Fang, Chunfang Gao

**Affiliations:** ^1^​ Department of Laboratory Medicine, Eastern Hepatobiliary Surgery Hospital, Second Military Medical University, Shanghai 200438, PR China; ^2^​ Department of Laboratory Medicine, 105th Hospital of PLA, Hefei 230031, PR China; ^3^​ Department of Clinical Laboratory, First Affiliated Hospital of Chinese PLA’s General Hospital, Beijing 100048, PR China

**Keywords:** hepatocellular carcinoma, hepatitis B virus, hepatitis B core antigen, preCore/Core, prognosis

## Abstract

The aim of this study was to explore the relationship between hepatitis B virus (HBV) core antigen (HBc) mutations and the post-operative prognosis of HBV-related hepatocellular carcinoma (HCC). In total, 98 patients suffering from HBV-related HCC and treated with surgery were enrolled, with a 48 month follow-up. The preCore/Core region of the HBV genome from tumour tissue (TT) and paired adjacent non-tumour tissue (ANTT) of these patients was sequenced, and a phylogenetic tree was reconstructed. The correlations between the viral features and evolutionary divergence of preCore/Core amino acid sequences from 67 paired TTs and ANTTs were analysed. Cox proportional hazard model analysis was applied for post-operative hazard risk evaluation. Phylogenetic analysis revealed that all of the sequences were ascribed to genotype C. The evolutionary divergence of amino acid sequences from matched TTs and ANTTs was significantly negatively correlated with serum and intrahepatic HBV DNA levels. Multivariate analysis showed that the HBc E77 mutation was associated with shorter overall survival, and HBc S87 and P156 mutations were independent risk factors for relapse. Furthermore, in contrast to with patients without the S87 mutation, no correlation was observed between serum HBV DNA and intrahepatic HBV DNA in HCC patients with the S87 mutation. Analysis of the intrahepatic sequence may advance our understanding of viral status; thus, it is useful for prognosis prediction for HBV-related HCC.

## Abbreviations

AFP, α-fetoprotein; ANTT, adjacent non-tumour tissue; AST, aspartate aminotransferase; DFS, disease-free survival; HBc, hepatitis B virus core antigen; HBV, hepatitis B virus; HCC, hepatocellular carcinoma; OS, overall survival; TNM, tumour node metastasis; TT, tumour tissue.

## Introduction

In China, hepatocellular carcinoma (HCC) ranked fourth and third for incidence and mortality, respectively, among all types of cancer during recent years [1]. Hepatitis B virus (HBV) infection is a major aetiological factor for primary liver cancer. Recent estimates attributed over 50 % of HCC cases worldwide and 65 % of HCC cases in China and the Far East to HBV infection [2, 3]. HBV contributes to HCC in both direct and indirect ways, and is implicated in both the occurrence and development of HCC [4]. Wild-type HBV X protein (HBx) was proved to induce liver cancer in transgenic mice directly in 1991 [5] and truncated HBx proteins rather than full-length HBx could effectively transform the immortalized liver cell line MIHA [6]. Similar to retrovirus, HBV genes could insert into host cancer genes such as those encoding telomerase reverse transcriptase (TERT), mixed-lineage leukaemia protein 4 (MLL4) and cyclin E1 (CCNE1) causing oncogenesis [7–9], and could exert effects on cell functions persistently. In addition, chimeric HBx-LINEs encoded by integrated viral genes could drive cell migration and invasion of tumour cell lines through the induction of epithelial mesenchymal transition [10] and the nuclear localization of β-actin [11]. Furthermore, wild-type or mutated viral proteins [HBx, HBV core antigen (HBc) and preS antigen] could also stimulate genomic instability [12–15] and directly affect cell functions and signal transduction pathways [16–19]. So, HBV plays a crucial role in both the formation and advanced stages of HCC. The HBV genome contains four overlapping ORFs encoding the surface antigen, the core antigen (capsid protein), the polymerase and a non-structural regulatory protein called X protein. The HBc and hepatitis B e antigen (HBeAg) sharing the same 149 aa are encoded by the same preCore/Core region in the HBV genome from an alternative upstream start codon [20]. The lack of a proofreading function for HBV polymerase causes a high mutation rate, estimated at one substitution in 10^3–6^ replication cycles, and is thought to play a substantial role in the progression of HBV-related HCC [21–23]. Plenty of studies have proved the essential relationship between HBV mutations and the development of HBV-related HCC [24–27]. Recent studies have focused on the prognostic value of these mutations in HBV-related HCC patients [28, 29]. For example the presence in liver tissue of A1762T/G1764A mutations within the basic core promoter region was an independent predictor for poor overall survival (OS) and disease-free survival (DFS) in HCC patients [30], and deletion in the preS region was also shown to be associated with shorter survival and a higher risk of recurrence after resection [30, 31]. Furthermore, mutations in HBc [32] and X protein [33] were also proved to be risk factors for inferior prognosis. Through sequencing of the preCore/Core region of the HBV genome from tumour tissue (TT) and paired adjacent non-tumour tissue (ANTT), this study was designed to extend current findings, and to disclose the association between HBc mutations and the recurrence and OS of HBV-related HCC patients after radical resection.

## Results

### Patient characteristics

A total of 98 HBV-related HCC patients were recruited. The baseline demographics, liver biochemistry tests, clinicopathological characteristics and virological data are listed in Table S1 (available in the online Supplementary Material). In total, 74 patients were diagnosed to have recurrent HCC and 48 patients died during follow-up. The overall cumulative recurrence rates of HCC were 58.2 and 76.5 % at 12 and 24 months, respectively. The overall cumulative survival rates were 80.6 and 55.1 % at 12 and 24 months, respectively.

### Phylogenetic tree building and genotyping

**Fig. 1.**
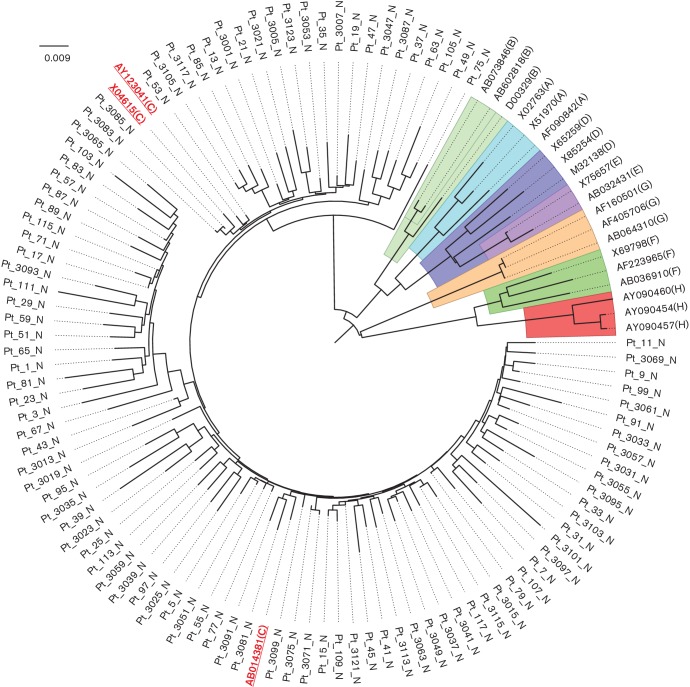
Neighbour-joining tree of preCore/Core sequences obtained from ANTTs of the 98 HBV-related HCC patients. Reference sequences of preCore/Core from HBV genotypes A–H are highlighted with coloured backgrounds, and preCore/Core reference sequences (AB014381, AY123041, X04615) of genotype C are underlined and highlighted by red text (scattered among the patient preCore/Core sequences).

Through evolutionary analyses (Fig. 1) and online genotyping, 98 preCore/Core sequences obtained from ANTTs in 98 HBV-related HCC patients were ascribed to genotype C. It is intriguing that no sequences were genotype B, which is the second most prevalent genotype in China.

### Evolutionary divergence analysis of preCore/Core and clinical implications

**Fig. 2.**
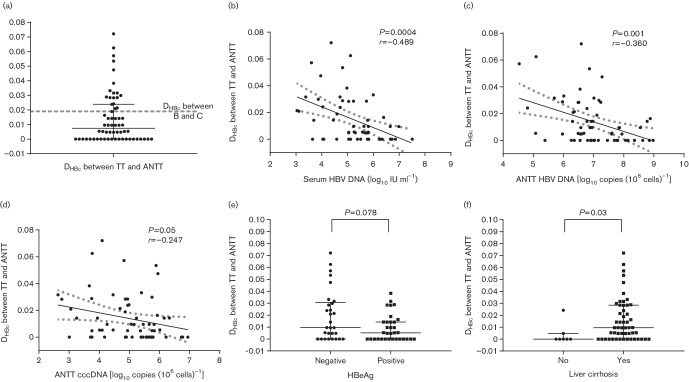
Distribution of *D*_HBc_ between paired TT and ANTT samples and its correlation with viral characteristics. (a) Distribution of *D*_HBc_ between paired TT and ANTT samples. The dotted line represents the *D*_HBc_ between genotypes B and C. (b,c,d) Correlation of *D*_HBc_ between paired TT and ANTT samples with serum HBV DNA levels (b), ANTT HBV DNA levels (c) and ANTT cccDNA levels (d).(e,f) Comparison of *D*_HBc_ from paired TT and ANTT samples between HBeAg positive and negative HBV-related HCC patients (e) and between HCC patients with or without liver cirrhosis (f).

With the Poisson model, the median evolutionary divergence of the intact preCore/Core amino acid sequence (*D*_HBc_) between 67 pairs of TT and ANTT samples was 0.009592 (0.0001–0.029165), smaller than the *D*_HBc_ (0.019) between genotypes B and C (Fig. 2a). Furthermore, the *D*_HBc_ between 24 pairs of TT and ANTT samples was zero, which means that no divergence was found in preCore/Core sequence between paired TT and ANTT samples in these patients. In addition, the *D*_HBc_ between paired TT and ANTT samples was found to significantly negatively correlate with serum HBV DNA levels (*P*<0.01, Fig. 2b), intrahepatic HBV DNA levels (*P*<0.01, Fig. 2c) and cccDNA levels (*P* *=*0.05, Fig. 2d). The *D*_HBc_ between paired TT and ANTT samples was also higher in HCC patients with cirrhosis (*P* *=*0.03, Fig. 2f) and HBeAg-negative HCC patients (*P* *=*0.078, Fig. 2e). Paired preCore/Core amino acid sequences from TTs and ANTTs were also subjected to phylogenetic analysis and 30 pairs of amino acid sequences were located in the same or the nearest clade in the phylogenetic tree (Fig. S1).

### Comparison of mutations in the preCore/Core between TTs and ANTTs

**Fig. 3.**
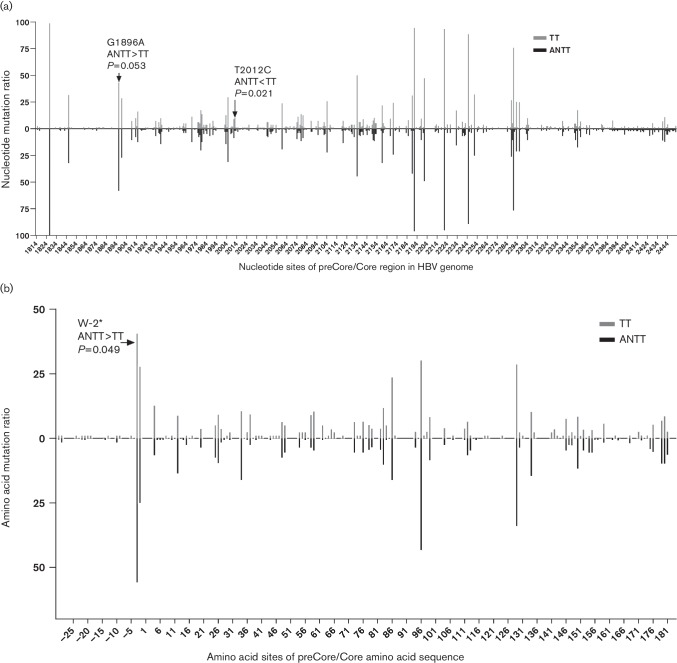
Comparison of the mutation distribution of preCore/Core in TTs and ANTTs. Nucleotide mutation (a) and amino acid mutation (b) distribution in preCore/Core of HBV originating from TTs and ANTTs of HBV-related HCC patients.

The distribution of nucleotide mutations and amino acid mutations is presented in Fig. 3(a, b). The mean mutation ratio of the preCore/Core in TTs and ANTTs was 2.3 and 2.2 %, respectively. No significant difference was found in the total mutation ratio for either nucleotide mutations or amino acid mutations between TTs and ANTTs, and only the nucleotide T2012C mutation (Fig. 3a) and abolishment mutation in amino acid preCore W-2 (Fig. 3b) have higher occurrence in ANTTs. In total, 30 of preCore/Core sites in TTs and 29 sites of preCore/Core sites in ANTTs were found to have a mutation ratio of greater than 5 %. The five most frequent mutations were preCore W-2Stop (56.1 %), I97L/F (43.6 %), P130T/Q/S (34.3 %), preCore G-1D (25.3 %) and S87G/R/N (16.5 %) in ANTTs, as well as in TTs (the corresponding mutation ratios were 40.8, 30.4, 28.9, 28.0 and 23.9 %, respectively; Fig. 3b).

### Clinicopathological parameters and preCore/Core mutations associated with prognosis in HCC patients

The Cox proportional hazard model was used to assess the relationship between clinicopathological parameters and mutations in preCore/Core associated with OS (Table 1) after surgical resection of HBV-related HCC patients. Univariate analysis showed that total bilirubin ≤14 µmol l^−1^, α-fetoprotein (AFP) >400 ng ml^−1^, tumour size >5 cm, tumour cell thick trabecular arrangement, portal vein tumour thrombus, higher tumour node metastasis (TNM) stage, and HBc mutations in E77, P79, E83, L84 and S87 were associated with shorter OS. Moreover, multivariate analysis showed that higher TNM stage and HBc mutation in E77 were associated with shorter OS.

**Table 1.** Univariate and multivariate analysis of clinicopathological parameters and HBc mutations for OS in HBV-related HCC patients

Parameter	No. of patients	Median of OS (range)	Univariate analysis	Multivariate analysis
			HR (95 % CI)	Adjusted HR (95 % CI)*
**Tbil (μmol l^−1^)**				
≤14	43	24.0 (12.3–27.4)		
>14	55	24.6 (19.0–34.1)	0.529 (0.299–0.937)†	
**AFP (ng ml^−1^)**				
≤400	49	24.9 (18.0–36.8)		
>400	48	21.8 (12.4–26.3)	1.953 (1.094–3.486)†	
**Largest tumour size (cm)**				
≤5	29	30.0 (24.0–36.8)		
>5	64	21.6 (11.7–25.5)	2.704 (1.331–5.491)‡	
**Tumour cell morphological classification**				
Thin trabecular	21	25.4 (22.0–36.8)		
Thick trabecular	70	24.0 (12.6–28.0)	4.241 (1.516–11.869)‡	
**Portal vein tumour thrombus**				
No	57	24.4 (15.3–29.3)		
Yes	23	13.2 (9.8–23.4)	2.483 (1.331–4.631)†	
**TNM stage**				
I–II	54	24.5 (16.0–32.4)		
III–IV	28	16.6 (9.5–24.9)	2.387 (1.258–4.528)‡	2.587 (1.286–5.204)‡
**HBc E77**				
E	86	24.0 (18.0–33.5)		
Q/D	6	12.7 (9.7–14.6)	6.021 (2.384–15.21)‡	4.302 (1.698–10.898)‡
**HBc P79**				
P	87	24.0 (15.3–33.1)		
Q	5	14.7 (9.3–16.8)	3.096 (1.097–8.736)†	
**HBc E83**				
E	87	24.0 (15.3–33.1)		
D	5	14.7 (9.3–16.9)	3.096 (1.097–8.736)†	
**HBc L84**				
L	81	24.0 (18.0–33.7)		
A/Q/S	11	12.9 (9.7–16.6)	3.601 (1.7–7.628)‡	
**HBc S87**				
S	75	24.7 (18.5–34.2)		
G/N/R	17	13.2 (9.97–18.6)	3.362 (1.682–6.72)‡	

HR, hazard ratio; Tbil, total bilirubin; A, alanine; D, aspartic acid; E, glutamic acid; G, glycine; L, leucine; N, asparagine; P, proline; Q, glutamine; R, arginine; S, serine.

*HR was adjusted for all other factors in the table.

† *P*<0.05.

‡ *P*<0.01.

Likewise, the Cox proportional hazard model was used to investigate the association between clinicopathological parameters and mutations in preCore/Core with tumour relapse of HCC patients after resection (Table 2). With univariate analysis, it was found that aspartate aminotransferase (AST) >40 U l^−1^, AFP >400 ng l^−1^, higher TNM stage and HBc mutations in E77, P79, E83, L84, S87 and P156 were high risk factors for recurrence of HCC. After adjusting for confounding variables, multivariate analysis showed that AST >40 U l^−1^ and HBc mutations in S87 and P156 were related to shorter DFS to a remarkable degree.

**Table 2.** Univariate and multivariate analysis of clinicopathological and HBc mutations for DFS in HBV-related HCC patients

Parameter	No. of patients	Median of DFS (range)	Univariate analysis	Multivariate analysis
			HR (95 % CI)	Adjusted HR (95 % CI)*
**AST (U l^−1^)**				
≤40	35	12.0 (7.5–30.8)		
>40	63	10.0 (2.9–17.7)	1.645 (0.998–2.712)†	3.424 (1.847–6.347)‡
**AFP (ng ml^−1^)**				
≤400	49	12.0 (8.0–32.0)		
>400	48	8.3 (1.8–13.8)	1.923 (1.21–3.056)†	
**TNM stage**				
I–II	54	12.0 (3.2–26.7)		
III–IV	28	7.4 (2.0–14.5)	1.698 (1.025–2.813)†	
**HBc E77**				
E	86	12.0 (5.7–23.3)		
D	6	2.6 (1.3–4.1)	5.388 (2.193–13.237)‡	
**HBc P79**				
P	87	12 (5.2–23.0)		
Q	5	2.0 (1.27–3.2)	2.763 (1.107–6.901)†	
**HBc E83**				
E	87	12.0 (5.2–23.0)		
D	5	2.0 (1.27–3.2)	2.763 (1.107–6.901)†	
**HBc L84**				
L	81	12.0 (6.0–24.0)		
A/Q/S	11	3.2 (1.0–6.2)	3.92 (2.008–7.653)‡	
**HBc S87**				
S	75	12.0 (6.0–23.7)		
G/N/R	17	2.0 (1.2–9.3)	2.072 (1.151–3.728)†	2.362 (1.238–4.506)‡
**HBc P156**				
P	87	12.0 (4.6–23.1)		
S/T	5	3.2 (3.2–9.0)	3.300 (1.291–8.439)†	7.595 (2.666–21.643)‡

HR, hazard ratio; A, alanine; D, aspartic acid; E, glutamic acid; G, glycine; L, leucine; N, asparagine; P, proline; Q, glutamine; R, arginine; S, serine; T, threonine.

*HR was adjusted for all other factors in the table.

† *P*<0.05.

‡ *P*<0.01.

### Mutations in HBc and post-operative prognosis

**Fig. 4.**
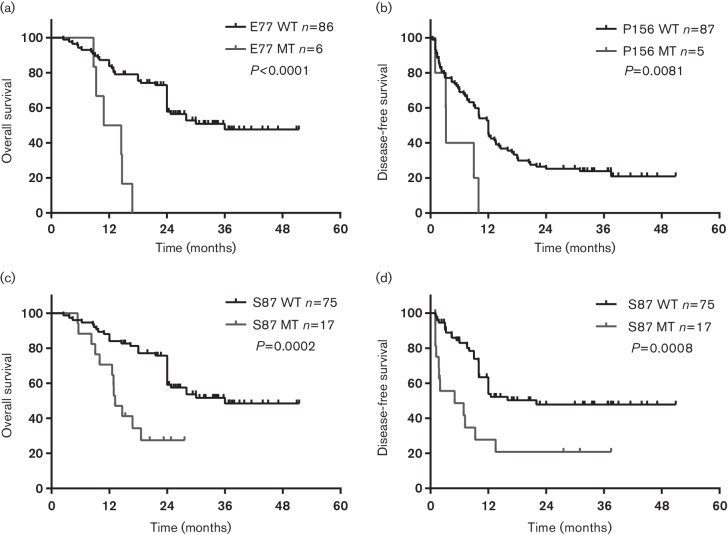
Association between post-operative survival and mutations in HBc. (a) Comparison of post-operative OS between patients with or without the E77 mutation in HBc. (b) Comparison of post-operative DFS between patients with or without the P156 mutation in HBc. (c, d) Comparison of post-operative OS (c) and DFS (d) between patients with or without the S87 mutation in HBc. MT, mutated type.

The Cox proportional hazard model showed that mutations in E77, S87 and P156 were important independent predictors for poor prognosis. Thus, Kaplan–Meier survival analysis was performed and there was significant association between the E77 mutation and OS (*P*=0.0001), and between the P156 mutation and DFS (*P*=0.0227) (Fig. 4a, b). The HBc S87 mutation was a significant predictor for DFS (*P*=0.0001) and OS (*P*=0.0004) (Fig. 4c, d).

### Mutations in HBc and clinicopathological factors

**Fig. 5.**
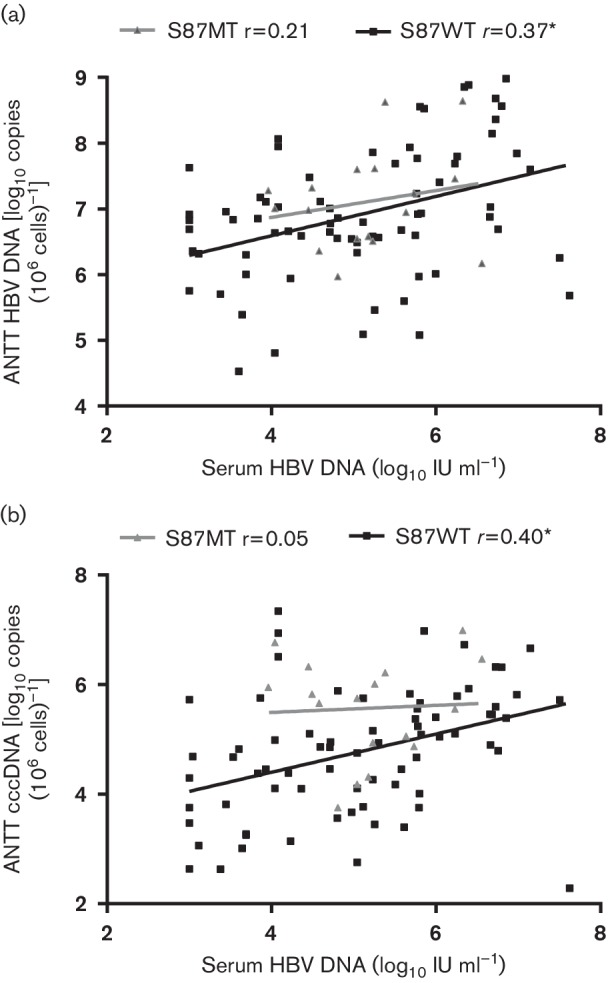
Correlation between serum HBV DNA levels and intrahepatic HBV DNA (a) and cccDNA (b) after stratifying for the presence of the HBc S87 mutation. *, *P*<0.05

Furthermore, the clinicopathological factors of HCC patients with or without HBc E77, S87 and P156 mutation were compared (Table S2), and patients with the E77 mutation displayed higher AST levels and larger tumour size. Patients with the S87 mutation were characterized by larger tumour size, higher TNM stage, and higher serum AFP and intrahepatocellular cccDNA levels. A previous study confirmed that phosphorylation of the serine of codon 87 of HBc facilitates core assembly [34]; thus, the correlation between serum HBV DNA and intrahepatocellular virus DNA of HCC patients was analysed, stratified for the presence of the HBc S87 mutation (Fig. 5a, b). In 17 HCC patients with the HBc S87 mutation, no correlation was identified between serum HBV DNA and intrahepatic HBV DNA and cccDNA. In contrast, serum HBV DNA was significantly correlated with intrahepatic HBV DNA and cccDNA in patients without the S87 mutation. In this regard, the presence of the S87 mutation impairs the assembly of the core particle and may contribute to the break off of the correlation between serum HBV DNA and intrahepatic HBV DNA. Only a lower albumin level was observed in patients with the HBc P156 mutation compared with patients without such a mutation. It is interesting that a higher mutation ratio in preCore/Core was observed in patients with the E77 mutation (*P*=0.002) or S87 mutation (*P*=0.004), and a moderate divergence in the mutation ratio of preCore/Core was found in patients with or without the P156 mutation (*P*=0.075).

## Discussion

HBc is the major component of the HBV core particle and the primary antigen targeted by the host immune system [35]. So, variation in preCore/Core plays an important role in the process of infection by affecting assembly capacity [34, 36, 37], immune epitope fluctuation [38–41] or virus pregenomic RNA packaging [42]. In recent years, more studies have disclosed the direct effect of HBc on cell apoptosis [43, 44], cell proliferation [45] and endoplasmic reticulum stress [46]. Therefore, the influence of HBc variation on the advancement of HCC may be more intriguing than previously thought, and more studies focusing on different areas or cohorts are still essential and irreplaceable. In the present study, most of the HBV-related HCC patients came from the Yangtze River Delta in eastern China. With phylogenetic analysis, it was unexpected that all of the preCore/Core sequences would be ascribed to genotype C, which is not consistent with previous studies [47, 48]. This discrepancy was indicative of a special evolutionary characteristic in the preCore/Core region that is inconsistent with the preS/S region, which is the decisive region for most HBV genotyping methods, and suggest that preCore/Core sequence of genotype C may be more adaptive in the HCC patients of eastern China. Moreover, inter-genotype combination incidence may also lead to discrepancy of genotype in different genes of the HBV genome [49].

Since a significant difference exists between TT and ANTT in HCC patients, the diversity of the preCore/Core sequence between virus in TT and ANTT was analysed, and no diversity was found in the substitution distribution and frequency between TTs and ANTTs in general, except for a moderate predominance in incidence of the G1896 mutation (preCore W-2Stop) in ANTTs, as reported previously [50, 51].

Further investigation showed that the median *D*_HBc_ between matched TTs and ANTTs was smaller than the *D*_HBc_ between genotypes B and C, and there were still 20 pairs of preCore/Core of TTs and ANTTs with the *D*_HBc_ greater than the *D*_HBc_ between genotype B and C, which means that there is still a certain degree of difference in the preCore/Core amino acid sequences between TTs and ANTTs. In addition, the relationship between the *D*_HBc_ of matched TT and ANTT with viral factors was assessed and there was significant negative correlation between the *D*_HBc_ of matched TT and ANTT with serum HBV DNA levels and intrahepatic HBV DNA and cccDNA levels. *D*_HBc_ may represent the complexity of intrahepatic virus and research on the HBV reverse transcriptase region revealed that the complexity of the virus quasispecies constitution could reflect the immune status [52, 53]. In this respect, a greater *D*_HBc_ reflects higher quasispecies complexity and always accompanies a strong immune response resulting in restrained HBV replication. However, a higher *D*_HBc_ may also be ascribed to the decreased replication capacity of a virus with higher gene complexity. The higher *D*_HBc_ in HCC patients with live cirrhosis may be indicative of persistent infection history and tendency to cirrhosis [54].

Currently, most studies have shown that the basal core promoter region double mutation was associated with an inferior prognosis for HCC [28, 30], while this study mainly focused on mutations in the preCore/Core. Multiple variable Cox regression showed that mutations in S87, E77 and P156 were associated with poor prognosis. The E77 and S87 mutations locate in the B cell epitope and P156 locates in the CD4^+^ T cell epitope [26, 35]. Previous research identified E77 within the cytotoxic lymphocyte (CTL) epitope and mutation in E77 could decrease epitope recognition preventing antigen presentation to CTLs [41]. In this respect, mutations in E77, S87 and P156, could affect epitope recognition, and cause immune escape, resulting in persistent infection. The S87 mutation was reported with a high prevalence in HCC patients [55, 56] and acute-on-chronic liver failure patients [57]. Mutation of S87 could also impair capsid assembly [34], and the irrelevance of serum HBV DNA and intrahepatic HBV DNA in patients with the S87 mutation observed in this study might be caused by impaired HBV assembly capacity. Consequently, more assembly-deficient HBc protein retention might have an influence on cell characteristics [43–45]. The P156 mutation lies within the C-terminus of HBc and close to a proline-rich loop (amino acids 128–136). The crystallographic structure of HBc suggests that this proline-rich loop serves important roles in HBV capsid localization, pregenomic RNA packaging and stabilization of the HBV capsid polymer structure [58]. Immune pressure results in escape mutations like HBc L60V and I97L [59], and when mutations, such as L60V and S87, impair core particle assembly [37, 40], HBc intrahepatic retention could activate downstream pathways, causing cell proliferation [45] and endoplasmic reticulum stress [46], similar to the intrahepatic retention of preS caused by deletion mutation [60]. This hypothesis may represent a carcinogenesis of HBc variation and should be further validated by experimental studies. Thus, mutations in HBc E77, S87 and P156 could impact on HCC advancement by affecting capsid stability, immune response and protein detention or localization.

There are limitations in this study. Firstly, the cohort is small and the follow-up time is short. A study with more patients and a longer follow-up time should be carried out to validate these significant HBc mutations. Secondly, laboratory work is indispensable in order to elucidate the way in which these important HBc mutations affect the progress of HCC. Finally, mutations in other regions of the HBV genome could also play roles in the prognosis of HCC patients and should also be included in the Cox proportional hazard model analysis in the future. However, with prediction values for more and more clinical or viral risk factors being assessed and validated, viral mutations such as drug-resistance mutations in the reverse transcriptase region and A1762T/G1764A double mutations in the basal core promoter region have been utilized in the clinical setting. So, the significant mutations in HBc, E77, S87 and P156 in our study may also have the potential to become prediction markers for the prognosis of HBV-related HCC patients after experimental validation and larger cohort validation.

In conclusion, preCore/Core evolutionary divergence between paired TT and ANTT was associated with virus replication and host immune status. The S87 and P156 mutations in HBc E77 were risk factors for poor prognosis, and analysis of HBc mutations could help with subgroup classification of HCC patients for personalized therapy.

## Methods

### Patients and samples

In total, 98 HBV-related HCC patients who received complete surgical resection between March 2007 and May 2008 at the Eastern Hepatobiliary Surgery Hospital, Shanghai, China, were recruited. TT and paired ANTT samples were collected and stored in a −80 °C refrigerator. The inclusion criteria comprised being serum hepatitis B surface antigen (HBsAg) positive for at least 6 months, having non-antiviral therapy before the operation, having had a complete resection of the tumour and pathological confirmation of HCC. The exclusion criteria included a history of liver transplantation and other malignancies, tumours of uncertain origin, human immunodeficiency virus co-infection, metastatic liver cancer, autoimmune liver diseases, drug-related liver diseases, alcoholic hepatitis and diagnosis with other causes of chronic liver diseases (such as hepatitis C, hepatitis D and so on) before enrolment. The study was supervised and approved by the Ethics Committee of the Eastern Hepatobiliary Hospital, with written informed consent obtained from all patients.

### Sanger sequencing and mutation analysis

HBV DNA in frozen TTs and ANTTs were extracted using the QIAamp DNA mini kit (QIAGEN), according to the manufacturer’s instructions. The target preCore/Core region in the HBV genome was amplified by PCR using two pairs of primers (Table S3). The PCR products were gel-purified and then sequenced by an ABI PRISM BigDye sequencing kit on an ABI 3500 genetic analyser (Applied Biosystems). All sequences were analysed in both forward and reverse directions. Sequence alignment was performed with mega 6.0 software [61]. The preCore/Core sequence of HBV genotype C (GenBank accession number: X04615) was used as a reference. The HBV genotypes were identified by an online genotyping tool (http://www.ncbi.nlm.nih.gov/projects/genotyping/formpage.cgi).

### Quantification of HBV cccDNA and HBV DNA in tissues

HBV cccDNA and HBV DNA concentrations in TTs and ANTTs were quantified using real-time PCR with TaqMan fluorescent probes (Fosun Diagnostics) using a previously described method [62]. The results for the cccDNA and HBV DNA were normalized to copies (10^6^ cells)^−1^.

### Routine laboratory tests

The liver biochemistry was measured by standard laboratory procedures with an automatic biochemistry analyser (Hitachi). HBV serological markers, including HBsAg, HBeAg, and antibody against HBsAg (HBsAb) and against HBeAg (HBeAb), were determined with a chemiluminescence microparticle enzyme immunoassay (Abbott). HBV DNA levels were determined with the Cobas Amplicor HBV monitor test (Roche Diagnostics), with a lower detection limit of 60 IU ml^−1^.

### Statistical analysis

The start codon of HBc was set as the origin site and the codons in the preCore region were labelled with negative serial numbers such as preCore W-2 or G-1. Amino acid mutation frequency was compared between TT and ANTT with the χ^2^ test. The phylogenetic tree was reconstructed according to the intact 92 preCore/Core sequences from 98 ANTTs with mega 6.0 software using the neighbour-joining method [63]. The preCore/Core sequence of 23 reference HBV genomes (GenBank accession numbers: X02763, X51970, AF090842, D00329, AB073846, AB602818, X04615, AY123041, AB014381, X65259, M32138, X85254, X75657, AB032431, X69798, AB036910, AF223965, AF160501, AB064310, AF405706, AY090454, AY090457, AY090460) from the GenBank database were also included in the phylogenetic tree. The D_HBc_ between 67 pairs of TT and ANTT was estimated using the Poisson correction model [64]. Survival curves were generated using the Kaplan–Meier method, and the log-rank test was used for comparison of the curves. Multivariate survival analysis was carried out using the forward stepwise Cox proportional hazards model. A threshold of *P*<0.05 was considered as statistically significant. All statistical analyses were performed with spss software (version 18.0).

## Supplementary Data

827 Supplementary File 1
