# Supplementary material for: Hepatitis B virus core antigen mutations predict post-operative prognosis of patients with primary hepatocellular carcinoma
Source: J Gen Virol. 2017 Jun 22;98(6):1399–409. doi: 10.1099/jgv.0.000790 (PMC5656792; doi:10.1099/jgv.0.000790)
Supplement: Supplementary File 1 [file jgv-98-1399-s001.pdf]

Supplementary Table 1 Demographics and clinicopathologic characteristics of HBV related HCC patients (n=98)

Parameter		Median (Range) or No.of patients (%)	Missing
Age (years)		50 (43.2-55.2)	
Tbil (μmol/L)		14.8 (12.5-18.4)	
Dbil (μmol/L)		5.4 (4.4-7.1)	
Alb (g/L)		41.1 (38.8-43.9)	
ALT (U/L)		44.4 (30.9-60.5)	
AST (U/L)		49.55 (37.4-64.6)	
GGT (U/L)		77.5 (47.3-142.2)	
ALP (U/L)		93.5 (77.3-126.8)	
AFP (ng/ml)		372.7 (17.1 to >1210)	
CA199 (U/l)		29.45 (17.6-44.7)	
RBC (10^9/L)		4.61 (4.37-4.87)	
HGB (g/L)		142 (135-151)	
PLT (10^9/L)		143 (96.5-196.5)	
PT (Second)		12.2 (11.7-12.9)	
Largest tumor size (cm)		8.1 (5.1-12.6)	
Serum HBVDNA (log_{10} IU/ml)		5.11 (4.08-5.81)	
Gender	Female	13 (13.3%)	
	Male	85 (86.7%)	
HBsAb	Negative	91 (92.9%)	1
	Positive	6 (6.1%)	
HBeAg	Negative	50 (51%)	1
	Positive	47 (48%)	
HBeAb	Negative	44 (44.9%)	1
	Positive	53 (54.1%)	

Tumor number	Multiple	27（27.6%）	5
	Single	66（67.3%）	
	None	48（49%）	
Tumor capsule	Partial	26（26.5%）	
	Intact	24（24.5%）	
Liver cirrhosis	No	11（11.2%）	5
	Yes	82（83.7%）	
HBV genotype	C	97（99%）	
	B/C	1（1%）	
TNM stage	I	26（26.5%）	16
	II	28（28.6%）	
	III	28（28.6%）	

Abbreviations: AFP, α-fetoprotein; Alb, albumin; ALP, alkaline phosphatase; ALT, alanine aminotransferase; AST, aspartate aminotransferase; CA199, carbohydrate antigen 199; Dbil, direct bilirubin; GGT, γ-glutamyltransferase; HBV, hepatitis B virus; HBeAb, hepatitis B e antibody; HBeAg, hepatitis B e antigen; HBsAb, hepatitis B surface antibody; HBsAg, hepatitis B surface antigen; HGB, Hemoglobin; RBC, red blood cell; PLT, platelet; PT, prothrombin time, Tbil, total bilirubin;

Supplementary Table 2. Comparison of clinicopathological factors between HCC patients with or without mutation in HBc E77, S87 and P156.

Amino acid site	E77			S87			P156		
Amino acid mutation	E (n=86)	Q/D (n=6)	*P*	S (n=75)	G/N/R (n=17)	*P*	P (n=87)	S/T (n=5)	*P*
Alb (g/L)	40.8 (39.2-44)	41.5 (37.5-42.5)	0.694	41.2 (38.6-44.3)	40.4 (39.7-41.8)	0.452	41.2 (39.6-44.1)	35.1 (34.6-40.7)	0.041*
AST (U/L)	48.5 (37.3-62.4)	64.9 (57.4-70.7)	0.041*	49.3 (37.3-62.4)	56.1 (38.4-81.6)	0.418	50.2 (37.6-68.0)	36.9 (34.6-38.4)	0.196
AFU (U/L)	33.5 (25.1-42.3)	33.5 (29.8-45.5)	0.370	32.1 (25.0-39.5)	45.1 (38.2-49.1)	0.000*	33.0 (25.1-41.75)	32.2 (29.1-33.4)	0.927
AFP (ng/ml)	298.4 (19.1-1210)	>1210	0.142	155.8 (14.8-1210)	1210 (417.6-1210)	0.017*	329.1 (19.8-1210)	417.6 (2.9-1210)	0.676
CEA (μg/L)	2.4 (1.6-3.6)	3.4 (3.1-5.2)	0.050	2.6 (1.7-3.8)	2.0 (1.2-2.9)	0.074	2.4 (1.7-3.65)	3.0 (1.3-3.2)	0.803
Serum HBVDNA (log_{10} IU/ml)	5.11 (4.08-5.83)	4.97 (4.21-5.66)	0.822	5.11 (4.04-5.86)	5.18 (4.58-5.63)	0.659	5.18 (4.08-5.86)	4.71 (4.08-4.81)	0.342
Tumor size (cm)	7.4 (5.0-11.2)	13.0 (13.0-14.5)	0.006*	6.6 (4.7-11.0)	12.0 (10.1-15.0)	0.001*	8.0 (5.0-12.0)	10.6 (5.0-13.0)	0.731

ANTT cccDNA (log_{10}copies/10^6cells)		4.94 (4.10-5.75)	4.96 (4.85-5.49)	0.496	4.84 (3.96-5.58)	5.75 (4.94-6.22)	0.007*	4.94 (4.10-5.75)	4.46 (3.75-5.24)	0.694
ANTT HBVDNA (log_{10}copies/10^6cells)		6.88 (6.55-7.61)	6.34 (5.15-6.88)	0.060	6.84 (6.39-7.62)	7.01 (6.55-7.46)	0.474	6.87 (6.49-7.60)	6.92 (6.30-7.01)	0.786
Amino acid mutation ratio in HBc (%)		2.36 (1.43-3.12)	4.95 (3.66-5.19)	0.002*	2.36 (1.42-3.12)	2.84 (2.37-5.19)	0.004*	2.36 (1.43-3.30)	5.19 (2.36-5.21)	0.075
Tumor number	Multiple	25	1	0.668	17	9	0.034*	26	0	0.316
	Single	59	5		56	8		59	5	
TNM Stage	I-II	47	5	0.660	45	6	0.007*	47	5	0.157
	III-IV	27	1		17	11		28	0	

Abbreviations: Alb, albumin; AFP, α-fetoprotein; AFU, α-fucosidase; AST, aspartate aminotransferase; CEA, carcino embryonic antigen; ANTT, adjacent non tumor tissue; D, Aspartic acid; E, Glutamic acid; G, Glycine; N, Asparagine; P, Proline; Q, Glutamine; R, Arginine; S, Serine; T,Threonine. ** $P<0.05$.

Supplementary Table 3. Primer pairs used in amplifying and sequencing the HBc and preC regions.

Name	Position	Direction	Sequence (5'-3')
C4	1606-1624	Forward	GCATGGAGACCACCGTGAA
D4	2388-2367	Reverse	GGCGAGGGAGTTCTTCTTCTAG
E5	2311-2332	Forward	CCCTATCTTATCAACACTTCCG
F5	476-457	Reverse	GACAAACGGGCAACATACCT

Supplementary Figure 1. Neighbor-Joining Tree of preCore/Core amino acid sequences obtained from paired TTs and ANTTs of the 67 HBV-related HCC patients.

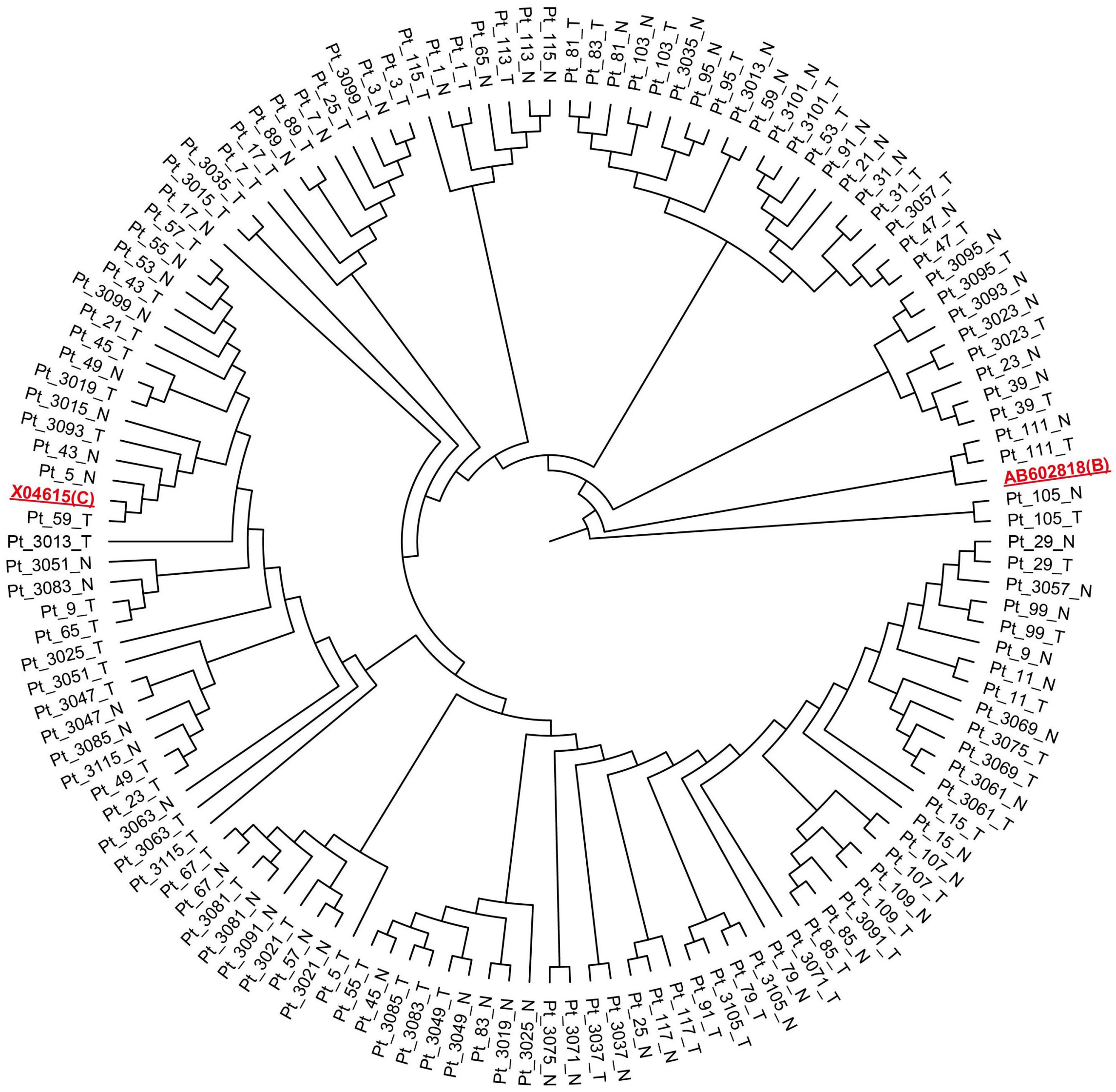

The preCore/core amino acid sequences from TTs and ANTTs were ended with T and N respectively.
